# New insights into X‐linked adrenal hypoplasia congenita from a novel splice‐site variant of NR0B1 and adrenal CT images

**DOI:** 10.1002/mgg3.2171

**Published:** 2023-04-28

**Authors:** Yuqing Jiang, Huifang Peng, Rui Zhao, Yina Chang, Jie Liu, Liujun Fu, Liping Li, Yujin Ma, Wei Li, Hongwei Jiang

**Affiliations:** ^1^ Henan Key Laboratory of Rare Diseases, Endocrinology and Metabolism Center The First Affiliated Hospital, and College of Clinical Medicine of Henan University of Science and Technology Luoyang 471003 China; ^2^ State Key Laboratory of Stem Cell and Reproductive Biology, Institute of Zoology Chinese Academy of Sciences Beijing 100101 China

**Keywords:** CT image change of adrenal, infertility issue, minigene, *NR0B1* (nuclear receptor subfamily 0 group B member 1), splice‐site variant, X‐linked AHC

## Abstract

**Background:**

X‐linked adrenal hypoplasia congenita (AHC) is a rare disorder, often manifesting as primary adrenal insufficiency (PAI) and hypogonadotropic hypogonadism (HH), and caused by variants of *NR0B1*, most of which are frame‐shifting variants, and few splice‐site variants.

**Methods and Results:**

Here, a novel splice‐site variant of *NR0B1* (NM_000475.4), c.1169‐2A>T (patient 1), and a stop‐loss variant of *NR0B1* c.1411T>C (patient 2) are described in this study. We perform minigene assays for the splice‐site variant (c.1169‐2A>T) and determine that the variant causes exon 2 skipping. Moreover, the defect of NR0B1 protein may bring about the severe phenotype of the patient. Through 8 years of follow‐up, we compare the CT images from 8 years ago with the latest image, and observe the CT image change of adrenal in patient 2 (from the increased thickness of adrenal to adrenal atrophy).

**Conclusion:**

X‐linked adrenal hypoplasia congenita is produced by variants of *NR0B1*. We report a case that presents a novel splice‐site variant, which has been verified that it could lead to the exon 2 skipping in the RNA splicing progress. Moreover, we report the adrenal CT image change of patient 2, which has never been referred to before, and expand the spectrum of X‐linked AHC characteristics.

## Graphical abstract

X‐linked adrenal hypoplasia congenita is produced by variants of NR0B1. We identified a novel splice‐site variant and determined that the variant caused the skipping of Exon 2 in NR0B1 during the splicing process via minigene assays. In addition, during the 8‐year follow‐up period, we identified a change in the size and morphology of the adrenal glands in one of the patients by CT images, which provided new information of the AHC patients. We also proposed that the adrenal atrophy may experience a dynamic process. Then the CT image of nonatrophy adrenal should not be neglected if a patient was considered as X‐linked AHC disease.

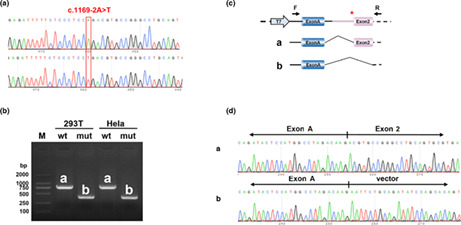

## 1 INTRODUCTION

Variants of *NR0B1* (OMIM* 300437) give rise to both adrenal hypoplasia congenita (AHC) and hypogonadotropic hypogonadism (HH). AHC (OMIM# 300200) is a rare disorder with an X‐linked pattern and is characterized by adrenal insufficiency in early life. Salt‐loss, vomiting, hyperpigmentation of the skin, insufficiency of cortisol and aldosterone, increased serum potassium and decreased sodium concentration, and high adrenocorticotropic hormone (ACTH) level are part of adrenal insufficiency and emerge during the early life or childhood of the patients. *NR0B1* has been viewed as an “orphan” nuclear receptor, also named *DAX1* (dosage‐sensitive sex reversal), for the reason that there is no specific ligand being identified (Iughetti et al., 2019). It has two exons flanking a 3.4 kb intron, with most of the coding sequence contained in exon 1, and encodes a protein product of 470 amino acids. Exon 1 encodes the DNA binding domain (DBD) and part of the ligand‐binding domain (LBD), while the remaining portion of the LBD is encoded by exon 2 (Niakan & McCabe, 2005). *NR0B1* expresses in embryonic stem cells, steroidogenic tissues (gonads, adrenals), the hypothalamus, and the pituitary, and in all regions of the hypothalamic–pituitary–adrenal (HPA) axis as well as hypothalamic–pituitary‐gonad (HPG) axis, suggesting that *NR0B1* plays a crucial role in the normal development and function of these axes (Szeliga et al., 2021).

In this study, one patient presents with the typical symptoms of X‐linked AHC, including adrenal crisis, salt‐loss, HH, and so on. Another patient appears with late‐onset symptoms and a relatively mild phenotype, and their distinctive features are profiled. By whole exome sequencing, we find a novel splice‐site variant of *NR0B1* (c.1169‐2A>T) in patient 1, and patient 2 presents with a stop‐loss variant of *NR0B1* (c.1411T>C). We conduct a minigene assay, which suggests that the variant (c.1169‐2A>T) in *NR0B1* leads to the exon 2 skipping in RNA splicing progress. Moreover, we observe CT image change of adrenal in patient 2 spanning 8 years. We introduce the specific cases through molecular and clinical characteristics, further expanding the understanding of AHC phenotypes and molecular mechanisms.

## 2 MATERIALS AND METHODS

### 2.1 Ethics statement

The First Affiliated Hospital Ethics Committee of Henan University of Science and Technology has ratified this study. Patients and their parents have already signed the informed consent agreement.

### 2.2 Clinical characteristics and WES of the patients and their family

Genomic DNA is extracted from peripheral blood samples of these patients and their parents. The library is prepared. We next hybridize and catch the entire exome DNA and part of the DNA in the nontranslated zone, including the intron region adjacent to exons ±20 bp, via the SureSelect Human All Exon V6 kit (Agilent). “Next‐generation” sequencing technology is practiced for identifying variants. Then, the sequencing data are compared to the human reference genome (hg19/GRCh37). We use ANNOVAR to annotate the variants. The frequency of each variant is determined in 1000 Genomes, NHLBI Exome Sequencing Project and Exome Aggregation Consortium to get rid of common SNPs. SIFT, POLYPHEN2 and METASVM are used to predict the pathogenicity of *NR0B1* variants. The reference sequence of *NR0B1* (NM_000475.4) was derived from the GenBank database. The American College of Medical Genetics and Genomics (ACMG) standards and guidelines for interpreting genetic variants are committed to analyzing the diseases concerned. Assay results are verified by Sanger sequencing.

### 2.3 In vitro splicing assays

To disclose how the *NR0B1* c.1169‐2A>T variant affects splicing, wild type (wt) and mutant (mut) *NR0B1* minigenes are generated. We set up 2 sets of vectors for the minigene experiment. The primers 5′‐TAGGAGTGCCTCTCGCTTAT‐3′ and 5′‐GCAAACGCAGTCTTCCATAC‐3′, 5’‐CATGGAAGATCAAAGGACCT‐3′ and 5′‐GCAGGTTCCATGAAATTGCT‐3′, are used for the nested PCR, taking the normal people DNA as a template.

#### 2.3.1 Vector of the pcMINI‐C‐*NR0B1* ‐mut

5′‐GGTAGGTACCTTAATGACTATAAGAATTTT‐3′, 5′‐TGCAGAATTCTGG.

AATAAAATTATTTCTTT‐3′ primers and *EcoRI* and *KpnI* restriction enzymes are used for the construction of pcMINI‐C‐*NR0B1*‐mut. The “mut” is for mutant.

#### 2.3.2 Vector of the pcDNA3.1‐*NR0B1* ‐mut

Meanwhile, the primers of 5′‐GCTTGGTACCATGTTGAAGACGCTGCGCTTCGT‐3′ and 5′‐TGCAGAATTCTGGAATAAAATTATTTCTTT‐3′, *EcoRI* and *KpnI* restriction enzymes are used for the vector of pcDNA3.1‐*NR0B1*‐mut. The “mut” is for mutant.

#### 2.3.3 Cell transfection and analysis of minigene transcription

Vector/DNA Fragment is subjected to enzymatic digestion reaction at 37°C for 2 h, and then the target bands are detected and recovered by electrophoresis. After overnight ligation at 4°C, the DH5 competence state is transformed and a number of monoclonal colonies are randomly selected for identification after overnight incubation at 37°C. The identification method included colony/liquid PCR and Sanger sequencing. The recombinant vectors are transiently transfected into HeLa and 293T cell lines, respectively, and the transfection procedure are performed according to the liposome instructions, and the samples are collected after 48 h. Extraction of the total RNA from the cell samples, and the extraction method are performed according to the kit instructions. After measuring the concentration, cDNA is synthesized by reverse transcription with equal amounts of RNA. PCR amplification is performed using the flanking primers 5′‐GCTTGGTACCATGTTGAAGACGCTGCGCTTCGT‐3′ and 5′‐TGCAGAATTCTGGAATAAAATTATTTCTTT‐3′ on the minigene vector, and the transcribed bands of the amplified genes are detected by agarose gel electrophoresis, and each band is recovered separately for Sanger sequencing.

## 3 RESULTS

### 3.1 Clinical characteristics of the cases

**FIGURE 1:**
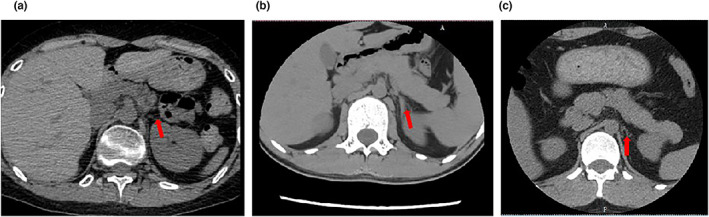
CT images the X‐linked adrenal hypoplasia congenita patients. Patient 1: adrenal atrophy (a). CT images Patient 2: adrenal with increased thickness (At the age of 17) (b) Patient 2: adrenal atrophy (At the age of 25) (c). The position of adrenal was labeled by the red arrow.

Patient 1, a 6‐year‐old male, was admitted to the endocrinology department with hyperpigmentation, nausea, vomiting, and was diagnosed with “adrenal deficiency” for 2 years. The above symptoms were alleviated by hydrocortisone 15 mg twice a day. The patient was the firstborn, born at full term, with a history of hypoxia (specific details unknown), and no history of special medication during the mother's pregnancy. At the age of 17, the patient had no development of secondary sex characteristics, and showed the low volume of the testis (3 mL on the left and 2 mL on the right), Tanner II of the breasts, no axillary and pubic hair. His basal biochemical measurements showed a high level of ACTH >2000 pg/mL (reference range: 7.2–63.3 pg/mL), and the normal rhythm of cortisol of 2.18 μg/dL at 0 am (reference range 0–6.7 μg/dL), 8.61 μg/dL at 8 am (reference range 4.2–24.8 μg/dL), 3.36 μg/dL at 4 pm (reference range 2.9–17.3 μg/dL), which was rendered by the treatment. The testosterone (TES) level showed 0.00 ng/mL, and the statistics of basal hormone levels were recorded in Table 1. The MRI scan of sella turcica showed no abnormalities. The abdominal CT revealed bilateral adrenal atrophy (Figure 1a). For the development of secondary sex characteristics, he was given testosterone undecanoate by intramuscular injection, 250 mg per month. He developed pubic hair, axillary hair, beard, and his stretched penile length (SPL) increased to 8 cm. Then the testosterone replacement therapy was discontinued and the human Chorionic Gonadotrophin (hCG) 2000 IU was administered by intramuscular injection, twice per week. He stopped it at the age of 19, and his axillary hair gradually disappeared. A GnRH (gonadotropin‐releasing hormone) test was adopted and revealed that pituitary and gonadal functions were defective (Table 2). At the age of 28, he was admitted to the Intensive Care Unit (ICU) with “shock” and hyperpigmentation, and was diagnosed with an “adrenal crisis.” The patient was given fluid replacement on admission to correct the water‐electrolyte imbalance, as well as intravenous hydrocortisone 200 mg per day. As the emergency was relieved, the patient was discharged with oral hydrocortisone, 15 mg twice a day to make up for the short half‐time of hydrocortisone, and hCG 2000 IU, twice a week.

**TABLE 1:** Basal hormone measurements, imaging examination, and variants of the X‐linked adrenal hypoplasia congenita patients.

	Patient 1 (17 years old)	Patient 2 (17 years old)	Patient 2 (25 years old)
ACTH 8:00 am (7.20–63.00 pg/mL)	>2000.00	>2000.00	1546.00
PRL	32.11 (1.61–18.77 ng/dL)	14.01 (2.50–17.00 ng/mL)	2.51 (2.50–17.00 mIU/mL)
FSH (1.50–12.50 mIU/mL)	3.60	9.10	14.70
LH (0.80–7.60 mIU/mL)	0.40	6.00	7.20
TES (262.00–1593.00 ng/dL)	0.00	1174.00	205.00
Adrenal atrophy (CT)	Yes	No	Yes
Increased adrenal thickness (CT)	No	Yes	No
Type of variant	c.1169‐2A>T	c.1411T>C	
Novel variant	Yes	No	

Abbreviations: ACTH, Adrenocorticotropic Hormone; CT, Computed tomography; FSH, Follicle‐stimulating hormone; LH, Luteinizing hormone; *NR0B1*, NM_000475.4; PRL Prolactin; TES, testosterone.

**TABLE 2:** Patient 1 GnRH stimulation test.

Time (min)	GnRH stimulation test	
	FSH mIU/mL	LH mIU/mL
0	<0.01	<0.01
30	<0.01	<0.01
60	<0.01	<0.01
90	<0.01	<0.01

Abbreviation: GnRH, gonadotropin‐releasing hormone.

Patient 2, a 17 year old male, had late onset and presented with fatigability and hyperpigmentation. The biochemical examination showed an extremely high level of ACTH >2000 pg/mL at 8:00 am and showed normal cortisol rhythm through the application of glucocorticoid replacement therapy. This patient did not complain of the symptoms of HH, and showed TES level 1174.0 ng/mL (reference range 262.0–1593.0 ng/mL), which reached the normal range. It was remarkable that the CT scan showed an adrenal with increased thickness (Figure 1b). The pituitary magnetic resonance found no abnormalities. He was diagnosed with “Addison's disease” and was given glucocorticoid replacement therapy, dexamethasone 0.75 mg, and prednisone 5 mg once daily. The unconventional replacement therapy aimed to suppress the high ACTH level. At the age of 25, he was admitted to the endocrinology department with recurrent fatigue. Hyperpigmentation of the skin was observed. Laboratory tests showed a high level of ACTH 1546.00 pg/mL, a relatively low level of TES 205.0 ng/dL. Adrenal scan and enhanced CT showed that bilateral adrenal glands were slender and reduced in size (adrenal atrophy) (Figure 1c), which drew our attention. We provided glucocorticoid replacement therapy, including dexamethasone 0.375 mg and prednisone 5 mg once daily.

### 3.2 WES of patients and their parents

**FIGURE 2:**
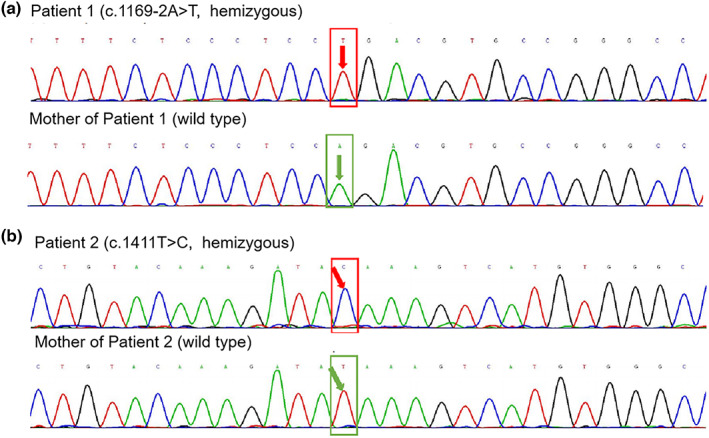
Sanger analysis of the X‐linked adrenal hypoplasia congenita patients. The reference sequence of *NR0B1* was derived from GenBank and the version number was NM_000475.4. Patient 1: Discovery of *NR0B1* gene c.1169‐2A>T variant, which was not detected in the sample of his mother. The red box represented the mutant base, and the green box represented the normal base (a). Patient 2: Discovery of *NR0B1* gene c.1411T>C, which was not detected in the sample of his mother. The red box represented the mutant base, and the green box represented normal base (b).

A novel variant was disclosed (c.1169‐2A>T), a splice‐site variant of the *NR0B1* gene, which was derived from patient 1 (Figure 2a) (den Dunnen et al., 2016). Meanwhile, the variant was not detected in the peripheral blood of patient 1's mother. This variant had never been recorded in the HGMD professional database and ClinVar database before and was judged to be “Pathogenic” (PVS1 + PS2 + PS3 + PM2).

The sample of patient 2 had a variant site in the exonic region of the *NR0B1* gene: c.1411T>C (Figure 2b), resulting in an amino acid change: p.Ter471GlnextTer18. The variant was a stop‐loss variant that had been reported by the HGMD professional database and ClinVar database for X‐linked AHC. According to ACMG guidelines, this variant was determined to be “Likely pathogenic” (PS2 + PM2 + PM4 + PP4) (den Dunnen et al., 2016; Yang et al., 2021). The variant was not found in his parents' samples.

### 3.3 In vitro splicing assays

We performed the minigene splicing assays to assess the effect of the *NR0B1* variant of c.1169‐2A>T of RNA splicing. Two sets of carriers (pcMINI‐C‐wt/mut and pcDNA3.1‐wt/mut) were built. The “wt” was for wild type. The “mut” was for mutant.

#### 3.3.1 Analysis of pcMINI‐C series

**FIGURE 3:**
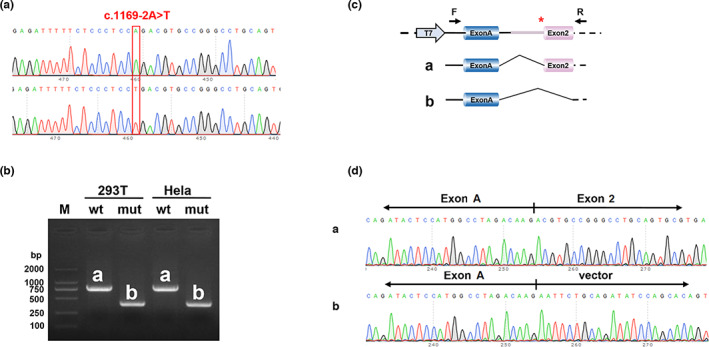
Analysis of pcMINI‐C vector assay for the *NR0B1* variant (c.1169‐2A>T): Sequencing diagram of minigene construction, “wt” at the top and “mut” at the bottom, the red box revealed the difference. THE “wt” was for wild type. The “mut” was for mutant (a). Agarose gel electrophoresis diagram of RT‐PCR transcription analysis, the results of RT‐PCR showed that the “wild type” was a single band of the expected size (773 bp) both in HeLa and 293T cells and was named the band “a.” The “mutant” was also a single band and was named the band “b.” THE “wt” was for wild type. The “mut” was for mutant (b). Diagram of spliced bands corresponding to sequencing results: the sequencing results showed that the wild‐type band “a” was a regular splicing band with the splicing mode of Exon A (192 bp)‐Exon 2 (402 bp), and the mutant band “b” was an abnormal splicing band with Exon 2 skipping and the splicing mode of Exon A (192 bp)‐vector (c and d). “T7” was for the promoter. Red * indicated variant location.

The results of RT‐PCR showed that the wild type was a single band of the expected size (773 bp) in HeLa and 293T cells and was named band “a” (Figure 3b). The mutant was also a single band and was named the band “b.” The sequencing results showed that the wild‐type band “a” was a regular splicing band with the splicing mode of Exon A (192 bp)‐Exon 2 (402 bp), and the mutant band “b” was an abnormal splicing band with Exon 2 skipping and the splicing mode of Exon A (192 bp)‐vector (Figure 3d).

#### 3.3.2 Analysis of pcDNA3.1 series

**FIGURE 4:**
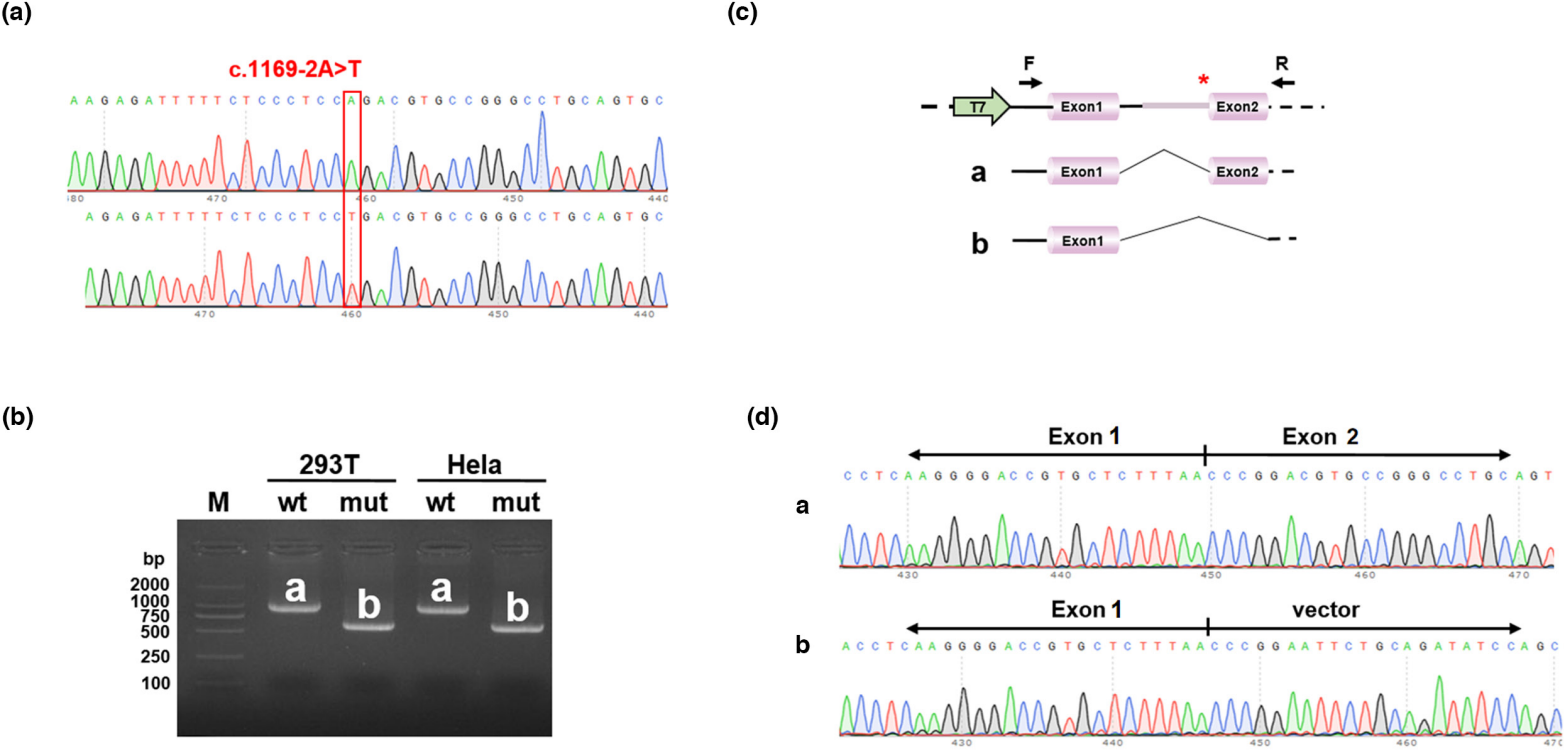
Analysis of pcDNA3.1 vector assay for the *NR0B1* variant (c.1169‐2A>T): Sequencing diagram of minigene construction with “wt” at the top and “mut” at the bottom, the red box revealed the difference. THE “wt” was for wild type. The “mut” was for mutant (a). Agarose gel electrophoresis plot of RT‐PCR transcription analysis: the RT‐PCR results showed that the wild type was a single band in HeLa and 293T cells, which was consistent with the expected size (969 bp) and named the band “a.” The mutant was also a single band and named the band “b.” THE “wt” was for wild type. The “mut” was for mutant (b); The sequencing results showed that the wild‐type band “a” was a regular splicing band with the splicing mode of Exon 1 (382 bp)‐Exon 2 (402 bp) and the mutant band “b” was an abnormal spliced band with Exon 2 skipping and the splicing mode of Exon 1 (382 bp)‐vector (c and d). “T7” was for the promoter. Red * indicates variant location.

The RT‐PCR results showed that the wild type was a single band in HeLa and 293T cells, which was consistent with the expected size (969 bp) and named the band “a” (Figure 4b). The mutant was also a single band and named the band “b.” The sequencing results showed that the wild‐type band “a” was a normal splicing band with the splicing mode of Exon 1 (382 bp)‐Exon 2 (402 bp), and the mutant band “b” was an abnormal spliced band with Exon 2 skipping and the splicing mode of Exon 1 (382 bp)‐vector (Figure 4d).

## 4 DISCUSSION

Many variants of *NR0B1* have been reported, mainly frame‐shifting variants and missense variants, with only five splice‐site variants (c.1169‐112_*17delinsTG, c.1169‐1G>A, c.1168+1_1168+20del, c.1168+1del, c.1168+1G>T verified in ClinVar database https://www.ncbi.nlm.nih.gov/clinvar/). The splice‐site variants would destroy normal splicing mode. More than a quarter of the alternative exons produced premature stop codons in their mRNAs, leading to the formation of truncated proteins or nonsense‐mediated decay and mRNA degradation (Hillman, Green, & Brenner, 2004). NR0B1 protein consisted of two conserved domains. As a conserved domain, DBD contained three repeat motifs of a leucine‐rich receptor‐binding motif, the so‐called LXXLL motif at the N‐terminus, and the LBD at the C‐terminus in NR0B1 protein (Fujieda et al., 2003). *NR0B1* had been identified as a negative coregulator of *SF1* transactivation, and the reason why disruption of *NR0B1* brought about adrenal, hypothalamic, and pituitary developmental defects, remained to be clarified (Iyer & McCabe, 2004). Loss of function of variants within the LBD abolished silencing function and ended in disabling to recruit corepressors, such as nuclear receptor corepressor (N‐CoR) to Steroidogenic Factor 1 (SF1) (Crawford, Dorn, Sadovsky, & Milbrandt, 1998). NR0B1 protein was localized both in the nucleus and cytoplasm, mainly the former, in which it directly combined with *SF1*, to suppress the transcription of *SF1*. The transcriptional activation function 2 (AF‐2) core sequence, also located at the C‐terminus and encoded by exon 2, and alternations of it would reduce the nuclear positioning frequency of SF‐1 (Xia, Huo, Wan, Wang, & Chang, 2018). We found a novel splice‐site variant in *NR0B1* (c.1169‐2A > T), eliminating the authentic splice donor site, which led to exon 2 skipping and eventually disrupted the structure and function of the protein. Exon 2 and the complete loss of the AF2, resulting in abnormal nuclear localization, were likely to elucidate the severe phenotype of patient 1 (experience of adrenal crisis). The defective splicing mode developed symptoms of AHC and HH within *NR0B1* (c.1169‐2A>T) of patient 1. Elevated or normal TES levels have been reported in infants with X‐linked AHC, but rarely reported in adults. Domenice et al. (Domenice, Latronico, & Brito, 2001) reported a 2‐year‐old Brazilian boy with *NR0B1* gene variant (c.430_431insG), who presented with precocious puberty, including pubic hair, enlarged penis and testes, and elevated TES levels. Combined with previous studies that reported patients presented with elevated or normal TES levels, high levels of ACTH might stimulate the synthesis of steroids in testicular Leydig cells, a process that is independent of the gonadotropin and leads directly to elevated testosterone levels in these infants. Patient 2 appeared to have a normal TES level. The direct protein–protein interactions between NR0B1 and SF1 controlled the SF1 steroidogenic activity for the downstream gene (such as WT1, AMH, STAR, CYP11.) and GnRH secretion by modulating NO synthase activation. Weaker interaction between the NR0B1 (c.1411T>C/p.Ter471GlnextTer18) protein and the wild SF1 protein may introduce SF1‐induced nNOS (an isoform of NOS) activation, which increases GnRH secretion (Chachlaki, Garthwaite, & Prevot, 2017). Therefore, it was likely that the altered mode of interaction between mutant NR0B1 and SF1 was the cause of unusually elevated androgen levels and the freeing from HH in patient 2. In addition, the HH was absent in some missense variants (p.Trp105Cys, p.Cys200Trp). The fact that these variants retained as much of their function (NR0B1 repression of SF1‐mediated transcription) as possible may explain the mild phenotype of the patients (Verrijn Stuart, Weiss, & Jameson, 2007).

During early adrenal development (50–52 days postconception), the fetal zone (FZ) and definitive zone (DZ) were the central component regions. The rudimentary adrenal gland developed about the 30th week of gestation, carrying zona glomerulosa (ZG)‐like and zona fasciculata (ZF)‐like components. Postnatally, there was a 50% reduction in adrenal weight within two weeks under the HPA axis (ACTH) action due to DZ differentiation, ZG, and ZF development. In the AHC patients, the DZ of the fetal adrenal gland was absent, while the FZ was cytomegalic and vacuolated, with the former responsible for cortisol secretion and the latter for dehydroepiandrosterone (DHEA) (Lotfi, Kremer, Dos Santos, & Cavalcante, 2018). The fact that *NR0B1* was significant in regulating progenitor cell development that could inhibit differentiation, expanding the progenitor cell population. When the *NR0B1* function defected, progenitor cells would differentiate into steroid cells prematurely, perform well in adrenal function in early life, and accumulate insufficient progenitor cells (Suntharalingham, Buonocore, Duncan, & Achermann, 2015). That was why symptoms of adrenal insufficiency were not usually observed after birth (neonatal period), but later (first two months of life or childhood), the adrenal function began to diminish due to shrinkage of progenitor cells, which had been verified in *NR0B1*‐KO mouse. Patient 2, with limited changes in the protein of NR0B1, had a late onset of disease and relatively mild symptoms. His adrenal CT image (at the age of 17 years old) showed the adrenal gland with increased thickness and showed adrenal atrophy (8 years later, at the age of 25 years old). This dynamic change in adrenal images had never been described before, which also extended the spectrum of characteristics of X‐linked AHC. The previous view was widely held that the atrophy and malfunction of the adrenal resulted from the defective *NR0B1*, which affected the development of the adrenal glands (Ouyang et al., 2021). Our study suggested that in some X‐linked AHC patients, the destruction of the adrenal glands was likely not to be the finished status but rather a gradual process.

For this patient 1, with the replacement therapy for HH, secondary sexual characteristics began to develop, and the patient's testosterone level was elevated quite satisfactorily. However, there was no significant improvement in the patient's testicular volume, which was synonymous with the previous studies that HH could be effectively improved by drug intervention. Few of these patients were fertile (Zheng et al., 2016). A previous study reported that an AHC patient with severe azoospermia accepted the TESE (testicular sperm extraction) to acquire sperms. Through ICSI (intracytoplasmic sperm injection) and a frozen–thawed singe‐embryo transfer, this patient and his wife gave birth to a healthy child (Frapsauce et al., 2011).

In conclusion, Whole exome sequencing was performed for patients with different onset times and characteristics, and finalized the diagnosis of X‐linked AHC by combining the clinical features of the patients. We identified a novel splice‐site variant and determined that the variant caused the skipping of exon 2 in *NR0B1* during the splicing process via minigene assays. In addition, during the 8‐year follow‐up period, we identified a change in the size and morphology of the adrenal glands in one of the patients by CT images, which provided new information of the AHC patients. We also proposed that the adrenal atrophy may experience a dynamic process. Then the CT image of nonatrophy adrenal should not be neglected if a patient is considered to have X‐linked AHC disease.

## AUTHOR CONTRIBUTIONS

Yuqing Jiang and Huifang Peng participated in designing the study and writing the paper. Rui Zhao, Yina Chang, Jie Liu, and Liujun Fu collected the data of all patents. Liping Li and Yujin Ma provided the reference and revised the manuscript. Hongwei Jiang and Wei Li guided the method and technology of this study. All authors contributed to this article and approved the submitted version.

## FUNDING INFORMATION

This work was supported by the Medical and Health Research Project in Luoyang (2001027A), the Science and Technology Development Plan Project in Henan Province (212102310780), the Joint Co‐construction Project of Henan Medical Science and Technology Research Plan (LHGJ20200585, LHGJ20210598).

## CONFLICT OF INTEREST STATEMENT

The authors declare that the research was conducted in the absence of any commercial or financial relationships that could be construed as a potential conflict of interest.

## ETHICS STATEMENT

This study was approved by the Ethics Committee of the First Affiliated Hospital of Henan University of Science and Technology. Informed consent was obtained from the patients and their parents. The clinical features, imaging examination, other clinical findings, and genetic variants of them were studied.

## Data Availability

Data are available on request from the authors.
